# A peroxynitrite decomposition catalyst prevents mechanical allodynia and NMDA receptor activation in the hind-paw ischemia reperfusion injury rats

**DOI:** 10.3892/etm.2013.1440

**Published:** 2013-12-09

**Authors:** KYUNG-HWA KWAK, HOON JUNG, JUN MO PARK, JIN-SEOK YEO, HYUNJEE KIM, HYUNG CHUL LEE, SUNG HYE BYUN, JONG-CHAN KIM, SUNG-SIK PARK, DONG GUN LIM

**Affiliations:** 1 Department of Anesthesiology and Pain Medicine, Kyungpook National University Hospital, Daegu 700-721, Republic of Korea; 2 Department of Anesthesiology and Pain Medicine, Keimyung University Dongsan Medical Center, Daegu 700-721, Republic of Korea

**Keywords:** ischemia/reperfusion injury, neuropathic pain, reactive oxygen species

## Abstract

The contributions of superoxide and nitric oxide to ischemia/reperfusion (I/R)-induced neuropathic pain have previously been demonstrated in an animal model that mimics the symptoms of complex regional pain syndrome type I (CRPS I). Targeting peroxynitrite, which is the product of their interaction, may provide effective treatments for I/R-induced neuropathic pain. In this study, the effect of the peroxynitrite decomposition catalyst FeTMPyP [5,10,15,20-tetrakis (N-methyl-4′-pyridyl)porphyrinato iron (III)], administered at doses of 1, 3 and 10 mg/kg via intraperitoneal injection 30 min prior to reperfusion, was evaluated in rats with chronic post-ischemic pain. The pain behavior of the rats was tested with a von Frey filament. Phosphorylation of N-methyl-D-aspartate (NMDA) receptors in the L4/6 section of the spinal cord was measured on the third day following reperfusion by western blotting. The rats treated with 3 or 10 mg/kg FeTMPyP demonstrated significant increases in their paw withdrawal thresholds and decreased levels of phosphorylated NMDA receptor subunit 1 compared with those of the vehicle group (all P<0.001). These findings suggest that nitrosative stress, specifically that associated with peroxynitrite, may be involved in the mechanical allodynia and central sensitization that are associated with CRPS I and may provide a rationale for CRPS I treatment strategies using peroxynitrite decomposition catalysts.

## Introduction

Complex regional pain syndrome (CRPS) is a neuropathic pain syndrome characterized by pain beyond the area of injury and impairment of the autonomic nervous system and motor function. The pathophysiological mechanisms leading to neuropathic pain in CRPS are considered to be complex and multifactorial, and certain aspects of the mechanism(s) remain to be elucidated (1).

A considerable amount of evidence implicates oxidative stress in the pathophysiology of CRPS type I (2,3). Eisenberg *et al*(2) demonstrated significant increases in the quantities of lipid peroxidation products and the antioxidant parameters in the serum and saliva of patients with CRPS I. It has also been observed that vitamin C, an antioxidant, reduces the prevalence of CRPS in humans following wrist fractures (3). Consistent with the clinical data, Coderre *et al*(4) demonstrated that painful hypersensitivity was reduced by free-radical scavengers and antioxidant therapy in animals with chronic post-ischemic pain (CPIP), which is a model of CRPS I. As an animal model for CRPS I, rats with CPIP display several symptoms that mimic human CRPS, including edema and chronic mechanical and cold allodynia with no direct nerve injury (4). Furthermore, the direct contributions of superoxide (O_2_^•−^) and nitric oxide (NO) were demonstrated as allopurinol, which inhibits xanthine oxidase (XO)-mediated superoxide production, or superoxide dismutase (SOD) and *N*-nitro-L-arginine methyl ester (L-NAME), which remove O_2_^•−^ and inhibit the synthesis of NO, significantly reduced mechanical allodynia in CPIP model rats (5).

Peroxynitrite (ONOO^−^), formed from the diffusion-controlled reaction of O_2_^•−^ with NO, is a highly toxic reactive oxygen species (ROS). It has been proposed that a number of the toxic effects of NO are due to the subsequent generation of ONOO^−^(6,7). ONOO^−^ is cytotoxic via several mechanisms, including the initiation of lipid peroxidation, the direct inhibition of mitochondrial respiratory chain enzymes, the inactivation of membrane sodium channels, the modifications of oxidative proteins, and the inhibition of antioxidant enzymes (7–9). Due to its toxic nature, ONOO^−^ may be involved in a number of inflammatory conditions (10,11), cardiovascular diseases (12) and neurodegenerative diseases (13).

Peroxynitrite has been implicated in several pathophysiological pain processes, such as thermal hyperalgesia associated with inflammation and nerve injury (14,15), opioid-induced hyperalgesia and antinociceptive tolerance (16,17), and spinal activation of the N-methyl-D-aspartate receptor (NMDAR) (18). Increased spinal NMDAR activity, as reflected by increased phosphorylation of NMDAR subunit 1 (NR1), is critically involved in the development of central sensitization as a basis of chronic pain (19–21).

However, although it is conceivable that nitroxidative stress may contribute to CRPS pathophysiology, the mechanism and significance of ONOO^−^ in CRPS have not yet been specifically explored. Thus, in the present study, the aim was to establish whether ONOO- was involved in the development of allodynia and NMDAR-mediated processes in a CPIP animal model that mimics the symptoms of human CRPS I. The effect of ONOO^−^ was examined by removing ONOO^−^ through the administration of a peroxynitrite decomposition catalyst, FeTMPyP [5,10,15,20-tetrakis(N-methyl-4′-pyridyl)porphyrinato iron (III)] pre-reperfusion and subsequently focusing on the preventative action of FeTMPyP in the ischemia/reperfusion (I/R) injury-induced CRPS I model.

## Materials and methods

### Animals

Male Sprague-Dawley rats (280–320 g) were used in the present study (Central Lab. Animal Inc., Seoul, Korea). Following their arrival, the rats were acclimated in their cages for three days prior to the experiment. All housing conditions and experimental procedures were conducted according to the National Institutes of Health (Bethesda, MD, USA) guidelines on laboratory animal welfare and under protocols approved by the Institutional Animal Care and Use Committee at the Kyungpook National University, Daegu, Korea.

### Induction of the CPIP model by hind paw I/R

Male Sprague-Dawley rats (n=20) were randomly allocated to one of five groups (n=4): i) Sham (sham surgery control group); ii) vehicle (CPIP control group); and CPIP rats treated with iii) 1, iv) 3 or v) 10 mg/kg FeTMPyP. All rats were treated at 30 min prior to reperfusion intraperitoneally. The CPIP model was induced as described by Coderre *et al*(4). Following induction of anesthesia with sodium pentobarbital, a Nitrile 70 Durometer O-ring (O-Rings West, Seattle, WA, USA) was placed around each rat’s left ankle joint for 3 h and then the O-ring was cut to allow reperfusion. The rats in the sham surgery control group underwent only anesthesia, similar to the CPIP animals, without an O-ring. For the CPIP control group, normal saline (the vehicle used to deliver FeTMPyP) was administered. The dosages of FeTMPyP (1, 3 and 10 mg/kg) were chosen based on previous publications (14,16,17). All the chemicals were from Sigma Chemical Co. (St. Louis, MO, USA) and freshly dissolved in normal saline immediately prior to the experiment.

### Hind paw mechanical allodynia

To assess the mechanical thresholds of the ipsilateral and contralateral hind paws, rats were acclimatized to a transparent acrylic box installed on a wire net for 15 min. A Dynamic Plantar Aesthesiometer (Ugo Basile, Comerio, Italy), operated in an automated von Frey device, was used for the measurement of mechanical allodynia. A von Frey filament (steel rod of 0.5 mm diameter) was pushed against the plantar surface of the hind paw with an ascending force of 0–50 g. The force at which the animal withdrew its paw was recorded. Animals were subjected to four consecutive trials with a minimum 10-sec interval and the average threshold was calculated. Rats were tested for mechanical allodynia prior to the I/R injury (baseline value) and on the third day following reperfusion when mechanical allodynia was at the maximum (5) by an observer blinded to the treatments.

### Western blot analysis

Animals were rapidly sacrificed. At the time of sacrifice, rats were deeply anesthetized by sodium pentobarbital (50 mg/kg, i.p.) and then perfused quickly with cold saline. The L4–6 section of the spinal cord was immediately harvested, separated into the left (ipsilateral) and right (contralateral) sides of the cord and frozen with liquid nitrogen. Subsequently, the spinal cord was dissolved in lysis buffer solution containing 20 mM Tris-HCl pH 8.0, 150 mM NaCl, 1 mM EDTA, 2 mM Na_3_VO_4_, 0.5 mM DTT, 10% glycerol, 1% Nonidet P-40 and protease inhibitor cocktail tablet (Roche Diagnostics, Mannheim, Germany). The samples were centrifuged at 13,800 × g for 20 min at 4°C, the supernatants were separated and the proteins were quantified by the Bradford method (Bio-Rad Protein Assay kit I; Bio-Rad, Hercules, CA, USA). Protein samples (50 μg) from each group were resolved in a buffer solution (0.1 M Tris-HCl, 10% glycerol, 2% SDS and 0.1% bromophenol blue). The samples were heated at 100°C for 5 min, loaded onto a 10% SDS-polyacrylamide gel electrophoresis gel, and then transferred onto nitrocellulose membrane (Whatman GmbH, Dassel. Germany). The membranes were blocked with Tris-buffered saline (50 mM Tris pH 7.4 and 10 mM NaCl) in 3% non-fat milk at room temperature for 1 h and incubated with a phosphorylated NRI (pNR1) antibody (Upstate Biotechnology, Inc., Temecula, CA, USA) at 4°C overnight. After washing with Tris-buffered saline (50 mM Tris pH 7.4, 10 mM NaCl), the membranes were incubated with anti-rabbit or anti-mouse horseradish peroxidase-conjugated secondary antibodies (Cell signaling Technology, Inc., Danvers, MA, USA) (1:2,000) for 1 h at room temperature prior to identifying the proteins with an ECL system (Amersham Biosciences, Buckinghamshire, UK). The densities of protein blots were quantified using LabWorks 4.5 software (Ultra-Violet Products, Cambridge, UK).

### Statistical analysis

The measured data are presented as the mean ± standard deviation. The data were analyzed with one-way analysis of variance, followed by post-hoc comparisons (Tukey’s HSD method) with SPSS, software, version 12.0 (SPSS, Inc., Chicago, IL, USA). P<0.05 was considered to indicate a statistically significant difference.

## Results

### Hindpaw mechanical allodynia

**Figure 1:**
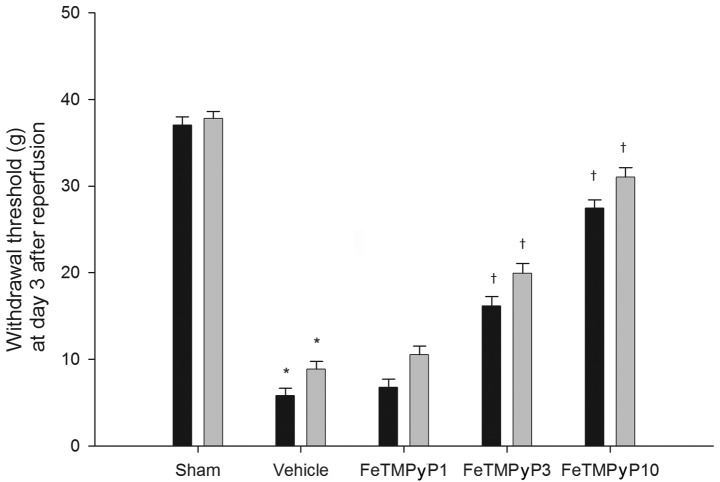
Effects of FeTMPyP on I/R injury-induced mechanical allodynia in the von Frey test. The von Frey test was performed on the third day following reperfusion. CPIP control rats (vehicle) exhibited significant reductions in ipsilateral (black bar) and contralateral (gray bar) paw withdrawal thresholds compared with the sham control rats (sham). FeTMPyP (3 and 10 mg/kg) was associated with significant protective effects. The data are presented as the mean ± SD. n=4 for all treatment groups. ^*^p<0.001 versus the sham group. ^†^P<0.001 versus the vehicle group. FeTMPyP, 5,10,15,20-tetrakis (N-methyl-4′-pyridyl)porphyrinato iron (III); FeTMPyP1, 1 mg/kg; FeTMPyP3, 3 mg/kg; FeTMPyP10, 10 mg/kg; I/R, ischemia/reperfusion; CPIP, chronic post-ischemic pain.

CPIP control rats exhibited significant reductions in their ipsilateral and contralateral paw withdrawal thresholds compared with those of the sham control rats. Treatment with 1 mg/kg FeTMPyP was not associated with a significant attenuation of the ipsilateral and contralateral paw withdrawal thresholds, whereas treatment with 3 or 10 mg/kg of FeTMPyP was associated with significant protective effects (Fig. 1).

### Measurement of pNR1

**Figure 2:**
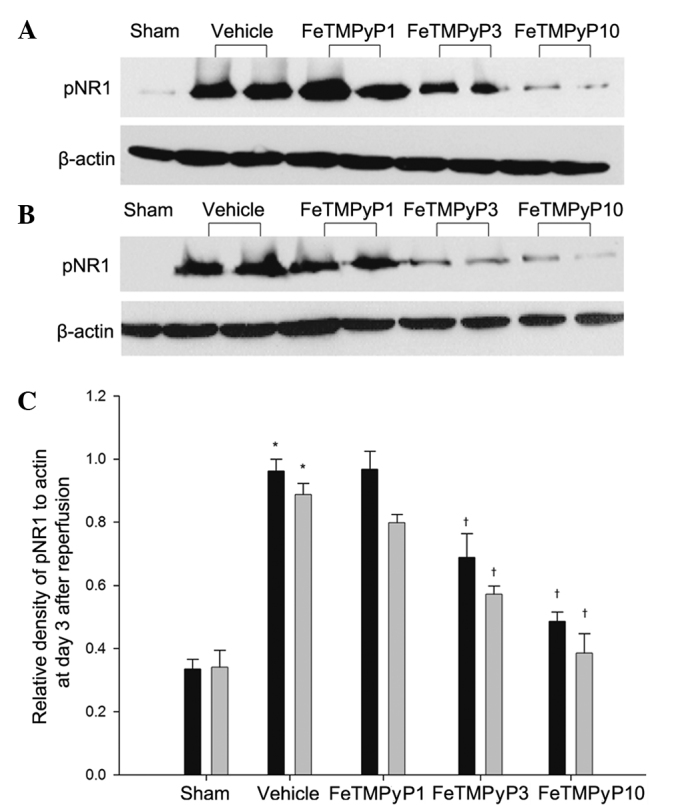
Changes in the relative density of pNR1 protein in the spinal cord (L4/6). Western blotting was performed to measure pNR1 expression levels on the third day following reperfusion in the (A) ipsilateral and (B) contralateral sides of the spinal cord. (C) Quantification of the pNR1 expression levels on the ipsilateral (black bar) and contralateral (gray bar) sides of the spinal cord based on western blot analysis. The data are presented as the mean ± SD. n=4 for all treatment groups. ^*^p<0.001 versus the sham group. ^†^P<0.001 versus the vehicle group. FeTMPyP, 5,10,15,20-tetrakis (N-methyl-4′-pyridyl)porphyrinato iron (III); FeTMPyP1, 1 mg/kg; FeTMPyP3, 3 mg/kg; FeTMPyP10, 10 mg/kg; pNR1, phosphorylated NMDAR subunit 1.

The degrees of ipsilateral and contralateral phosphorylation of NR1 were demonstrated to be significantly increased in the spinal cords of the CPIP control rats compared with those of the sham control rats. Treatment with 3 or 10 mg/kg FeTMPyP significantly decreased the ipsilateral and contralateral pNR1 levels. A degree of reduction was also observed in the 1 mg/kg FeTMPyP-treated group, but the change was not statistically significant (Fig. 2).

## Discussion

ONOO^−^ is well established as a very reactive species, inducing cytotoxicity in various diseases, but its involvement in the pain associated with CRPS is not well understood. Using a CPIP rat model exhibiting symptoms that resembled those of humans with CRPS, the results of the present study demonstrated that ONOO^−^ is a determinant of pain in this setting.

CRPS I is a chronic pain syndrome that occurs following relatively benign injuries, such as sprains, fractures, tissue trauma and ischemia, with no definable nerve lesion (22). The CPIP model was developed by Coderre *et al*(4) and was used to study the mechanisms that potentially underlie CRPS I. Coderre *et al*(4) demonstrated that 3-h I/R of the hind paw induced several symptoms that mimicked human CRPS I, including edema, as well as chronic mechanical and cold allodynia, with no direct nerve injury.

I/R injury is the tissue damage caused when the blood supply returns to a tissue following a period of ischemia. Prolonged ischemia leads to the accumulation of oxidases, which are enzymes that produce free radicals. The reintroduction of molecular O_2_ into ischemic tissue upon reperfusion leads to the overproduction of ROS. A cascade of ROS formation is initiated by the generation of O_2_^•−^, which is generated by XO. NO and O_2_^•−^ may react together to produce significant amounts of a much more oxidatively active molecule, ONOO^−^, which is a potent oxidizing agent that causes post-hypoxic cellular injury (23). A study has observed XO-mediated O_2_^•−^ production in a CPIP model and demonstrated that O_2_^•−^ and NO mediate I/R injury-induced chronic pain (5). O_2_^•−^ and NO are highly reactive and unstable (24); their reaction is ~3 times faster than the dismutation of O_2_^•−^ by SOD. Additionally, ONOO^−^ is a strong oxidant and is more stable than NO or O_2_^•−^(25). The stability of ONOO^−^ is sufficient to allow it to cross several cell diameters to reach target cells prior to becoming protonated and decomposing (26). Evidence supports the major involvement of ONOO^−^ in the development of tissue damage during inflammation (27,28), as well as in the pain of several etiologies (29).

Previous studies have provided concrete evidence implicating peroxynitrite in the development of certain aspects of chronic pain (30,31). Peroxynitrite has been reported to contribute to the development of chronic pain by means of peripheral and central actions. These studies identified that the peripheral formation of ONOO^−^ contributes to hyperalgesia by favoring the production of several proinflammatory cytokines and by increasing the production of prostaglandin E2. Peripheral administration of ONOO^−^ or ONOO^−^ precursors induces inflammatory hyperalgesia (14). In a neuropathic pain model, the daily administration of uric acid decreased ONOO^−^-mediated nitration in peripheral nerves and alleviated thermal hyperalgesia and Wallerian degeneration (15). These effects may also occur in CNS regions responsible for pain processing through the mediation of central sensitization. ONOO^−^ is considered to contribute to central sensitization through the alteration of NMDAR activation by nitrating proteins that are important in the maintenance of normal nociceptive processing, such as MnSOD (32,33), glutamate transporters (GTs) and glutamine synthase (GS) (34,35). The ONOO^−^-mediated nitration of MnSOD inactivates the enzyme, which results in increased O_2_ and ONOO^−^ levels, leading to enhanced postsynaptic neuronal responsiveness that contributes to central sensitization (18,29,32,33). Nitration of GLT-1 and GS disrupts glutamate homeostasis and increases glutamate neurotransmission, and the resulting signaling events underlie central sensitization. Glutamate is the primary endogenous ligand for the NMDAR. Extracellular glutamate concentrations have to be maintained low enough to terminate glutamate receptor activation. When GTs are nitrated by ONOO^−^, their inactivation results in increased glutamate concentrations and altered synaptic transmission (34). GS, which catalyzes the conversion of glutamate and ammonia to glutamine, is also inactivated via nitration by ONOO^−^(35).

In the present study, it was demonstrated that the ONOO^−^ decomposition catalyst FeTMPyP prevented NMDAR activation, suggesting another mechanism for ONOO^−^ in the spinal cord in addition to its ability to block mechanical allodynia. It is possible that I/R injury induces pathological responses in the CNS similar to those induced by mechanical or inflammatory nerve damage through ONOO^−^-induced nitrosative processes. Although the results suggest ONOO^−^ is potentially involved in mechanical allodynia and central sensitization, the site of action has not been identified as the drugs used were administered systemically. To this end, and as ONOO^−^ is produced in peripheral nociceptors and also in central nociceptors, it is likely that mechanical allodynia is associated with increased ONOO^−^ formation in peripheral and central pathways.

In effect, counteracting the damage associated with ONOO^−^ may be accomplished by decomposing ONOO^−^ and also by preventing the formation of ONOO^−^. In a previous study, in which the formation of SO and NO, as a precursor of ONOO^−^, was prevented in NMDA-mediated central sensitization using L-NAME and SOD in CPIP rats, it was indirectly observed that ONOO^−^ from these reactive species may be a causative factor in allodynia in CPIP rats, since the prevention of ONOO^−^ formation centers on inhibition of the formation of SO and NO. However, NO is often considered to be pro-nociceptive by generating nitrogen free radicals and causing vasodilatation, thereby facilitating inflammatory processes. NO-induced vasodilatation may relieve pain-producing vasospasm in the CPIP model. Thus, decomposing ONOO^−^ appears to be an improved strategy for reducing free radical-mediated toxicity as it preserves the positive effects of NO.

In conclusion, the results of the present study indicate that ONOO^−^ is critically involved in the pathogenesis of I/R injury-induced neuropathy and the formation of ONOO^−^ in the spinal cord in response to NMDAR activation, and that ONOO^−^ contributes to the development of central sensitization. A ONOO^−^ decomposition catalyst demonstrated a protective effect against CPIP, as evidenced by the improvement in mechanical allodynia and central sensitization. The present study supports the potential of ONOO^−^ as a novel target for pain management in CRPS patients.
